# A MLVA Genotyping Scheme for Global Surveillance of the Citrus Pathogen *Xanthomonas citri* pv. *citri* Suggests a Worldwide Geographical Expansion of a Single Genetic Lineage

**DOI:** 10.1371/journal.pone.0098129

**Published:** 2014-06-04

**Authors:** Olivier Pruvost, Maxime Magne, Karine Boyer, Alice Leduc, Christophe Tourterel, Christine Drevet, Virginie Ravigné, Lionel Gagnevin, Fabien Guérin, Frédéric Chiroleu, Ralf Koebnik, Valérie Verdier, Christian Vernière

**Affiliations:** 1 UMR Peuplements Végétaux et Bioagresseurs en Milieu Tropical (PVBMT), CIRAD, Saint Pierre, La Réunion, France; 2 Institut de Recherche pour le Développement, UMR Résistance des Plantes aux Bioagresseurs (RPB), IRD-CIRAD-UM2, Montpellier, France; 3 Institut de Génétique et Microbiologie, UMR 8621, Université de Paris-Sud, Orsay, France; 4 Institut de Génétique et Microbiologie, UMR 8621, CNRS, Orsay, France; 5 UMR Biologie et Génétiques des Interactions Plante-Parasite (BGPI), CIRAD, Montpellier, France; 6 UMR Peuplements Végétaux et Bioagresseurs en Milieu Tropical (PVBMT), Université de la Réunion, Saint Pierre, La Réunion, France; Virginia Tech, United States of America

## Abstract

MultiLocus Variable number of tandem repeat Analysis (MLVA) has been extensively used to examine epidemiological and evolutionary issues on monomorphic human pathogenic bacteria, but not on bacterial plant pathogens of agricultural importance albeit such tools would improve our understanding of their epidemiology, as well as of the history of epidemics on a global scale. *Xanthomonas citri* pv. *citri* is a quarantine organism in several countries and a major threat for the citrus industry worldwide. We screened the genomes of *Xanthomonas citri* pv. *citri* strain IAPAR 306 and of phylogenetically related xanthomonads for tandem repeats. From these *in silico* data, an optimized MLVA scheme was developed to assess the global diversity of this monomorphic bacterium. Thirty-one minisatellite loci (MLVA-31) were selected to assess the genetic structure of 129 strains representative of the worldwide pathological and genetic diversity of *X. citri* pv. *citri*. Based on Discriminant Analysis of Principal Components (DAPC), four pathotype-specific clusters were defined. DAPC cluster 1 comprised strains that were implicated in the major geographical expansion of *X. citri* pv. *citri* during the 20^th^ century. A subset of 12 loci (MLVA-12) resolved 89% of the total diversity and matched the genetic structure revealed by MLVA-31. MLVA-12 is proposed for routine epidemiological identification of *X. citri* pv. *citri*, whereas MLVA-31 is proposed for phylogenetic and population genetics studies. MLVA-31 represents an opportunity for international *X. citri* pv. *citri* genotyping and data sharing. The MLVA-31 data generated in this study was deposited in the *Xanthomonas citri* genotyping database (http://www.biopred.net/MLVA/).

## Introduction

Genotyping is a fast and powerful strategy to distinguish individuals and determine the genetic relationships between them. Genotyping has become a central and unifying approach in several research fields of microbiology, such as phylogenetics, taxonomy, population genetics and epidemiology [1]. Molecular epidemiology studies of pathogens are primarily achieved at two different spatial scales and with different objectives: (i) broad genotyping-based worldwide surveillance (i.e. global epidemiology [2]) and (ii) outbreak investigation at local or regional scales. MultiLocus Sequence Typing (MLST) targeting housekeeping genes has become increasingly popular for molecular epidemiology analyses of pathogenic bacteria [3]. However, its resolution for monomorphic pathogens is too low since they contain so little sequence diversity. Sequencing a few housekeeping gene fragments yields little or no polymorphism and fails to resolve the evolutionary patterns of such populations [4]. A large number of bacterial pathogens of agricultural importance are monomorphic [5]. In contrast to human-pathogenic bacteria [3], [4], very little is known about the population biology of plant-pathogenic bacteria even if some of them have a tremendous economic impact on agriculture [6].

New sequencing technologies easily generate nearly complete genome sequences and considerably facilitate the discovery and validation of new genetic markers, such as tandem repeats (TR) [7]. TR loci are among the most variable regions in bacterial genomes and, therefore, have the potential to resolve the genetic diversity of monomorphic pathogens [8]. TR copy number variation is mostly the result of slipped strand mispairing (slippage) during DNA replication [9]. Several factors have an impact on the variation rate at TR loci, such as: TR unit size and total size of the array, degree of sequence identity, the repeat's ability to form secondary structures, strand orientation, flanking sequences and the accuracy of DNA repair systems [8], [10]. MultiLocus Variable number of tandem repeat Analysis (MLVA) is a simple and robust method that has the potential to provide the necessary level of resolution for pathogens that cannot be appropriately genotyped by MLST, as in the case of *Bacillus anthracis*, *Yersinia pestis* and *Mycobacterium tuberculosis* [11]–[13]. Minisatellite-based MLVA of mycobacteria, also known as mycobacterial interspersed repetitive unit variable number of tandem repeats (MIRU-VNTR), showed the highest level of inter-laboratory reproducibility when compared to other genotyping techniques, such as spacer oligonucleotide typing (spoligotyping), amplified fragment length polymorphism (AFLP) or ligation-mediated PCR [14]. MIRU-VNTR has proved to be useful for assessing the evolutionary patterns of *M. tuberculosis* [15].

*Xanthomonas citri* pv. *citri*, a monomorphic gamma-proteobacterium, is the causal agent of Asiatic citrus canker (ACC), a major constraint in most citrus-producing areas worldwide [16]. As a result of its economic impact and the difficulties to control the disease, *X. citri* pv. *citri* has been listed as a quarantine organism in citrus-producing countries that are disease free or where the disease has been eradicated (e.g. Australia, New Zealand, South Africa, members of the European Union, North Africa, several US states). Moreover, *X. citri* pv. *citri* is listed as a dual-use organism in the European Union because of its potential use as a biological weapon (directive 394/2006 EC) [17].

Both *X. citri* pv. *citri* and its primary host genus (*Citrus*) originated from Asia [18], [19]. The pathogen's geographical expansion beyond Asia was first reported in the first half of the 20^th^ century [20]–[25]. A pathotype classification of *X. citri* pv. *citri*, which reflects differences in host range among strains, was proposed [26]. Some groups of strains (pathotypes A* and A^w^) are restricted to the highly susceptible Mexican lime (*Citrus aurantifolia*) and a few related species. However, genes governing host range restriction are different in pathotypes A* and A^w^ strains [27], [28]. In contrast, other strains (pathotype A) can infect nearly all *Citrus* species, as well as rutaceous-related genera [16]. Pathotype A* has been reported from several countries in Asia [26]. While pathotype A^W^ was first reported in Florida, it was subsequently found that these strains likely originated from India [29], [30]. Although pathotypes A* and A^w^ strains have a lower economic impact due to their narrow host range, they can cause severe damage to Mexican lime, as illustrated by the extensive cankers and dieback recently caused by A* Thai strains [31]. Pathotype A strains are currently prevalent in East Asia, the Indian Ocean region, South America and Florida [16]. In recent decades, globalization has drastically increased international movement of plants and plant products through trade and human travel. Consequently, the introduction of pests and pathogens in agricultural crops is increasing, in terms of both frequency and variability of geographical origins [32]. In the case of plant-pathogenic bacteria, a meta-analysis highlighted migrations (i.e. introduction from remote areas) as the major driving force behind emergence [6]. The recent introduction of *X. citri* pv. *citri* in several African countries where citrus canker had not been observed previously is a striking example of this type of migration [33]–[37].

Sequence polymorphism within *X. citri* pv. *citri* has been examined at nine housekeeping genes [38], [39]. The currently targeted housekeeping genes display extremely low polymorphism and do not provide sufficient resolution of *X. citri* pv. *citri* genetic diversity. In order to further our understanding of the pathogen's global epidemiology, it would be useful to develop alternative genotyping methods that include most desirable characteristics, such as maximal typeability (i.e. the proportion of strains that can be successfully genotyped at all loci [40]), reproducibility and inter-laboratory comparability. Various phenotypic and genotypic methods (including rep-PCR, AFLP and insertion sequence-based techniques) have been developed for typing worldwide *X. citri* pv. *citri* collections. However, these methods are time-consuming, not enough resolutive and/or sometimes unable to allow reliable inter-laboratory strain comparisons [29], [41]–[43]. A MLVA scheme targeting 14 short (≤7 bp long) TRs (MLVA-14) has been developed for *X. citri* pv. *citri* [29], [44]. Such markers with very high mutation rates have a very high discriminatory power but tend to produce datasets with a partially obscured phylogenetic signal, partly because of size homoplasy (i.e. the occurrence of genotypes that are identical by state but not by descent) [45]. Therefore, the MLVA-14 scheme is not well suited for evaluating phylogenetic relationships among epidemiologically-unrelated strains [29], [45] but was found very useful for outbreak investigations at small to medium spatio-temporal scales [46]. Remarkably, the significant correlation between genetic distances derived from independent sets of markers reveals a lack of frequent recombination among *X. citri* pv. *citri* strains and underscores the vast potential of genotyping to trace strains over time and space [29], [46].

The primary aim of the present study was therefore to develop and evaluate a robust MLVA scheme suitable for global studies on the epidemiology of *X. citri* pv. *citri* based on new TR loci with larger (≥10 bp) repeat unit sizes, as identified by whole genome sequence mining. We show that our strain collection, which is representative of the worldwide genetic and pathological diversity of *X. citri* pv. *citri*, is structured into four pathotype-specific clusters. A single cluster of strains, which was detected in nearly all regions of the world where Asiatic citrus canker has become established, was identified as the primary source of the major geographical expansion of *X. citri* pv. *citri* during the 20^th^ century. We further assessed the polymorphism level among epidemiologically related strains using two large strain collections associated with recent outbreaks in Réunion Island and Vietnam.

## Materials and Methods

### Bacterial strains, media and DNA extraction

Two hundred and sixteen strains of *X. citri* pv. *citri* isolated from several continents and representative of the worldwide genetic and pathological diversity were used (Tables S1 and S2). A “panel test” comprising 20 strains representative of the genetic diversity of *X. citri* pv. *citri* were used for preliminary primer screening (Table S1). All strains were stored as lyophilisates and/or at −80°C (Microbank, Pro-lab, Round Rock, TX, USA). Two additional strain collections were analyzed (i) 50 epidemiologically related *X. citri* pv. *citri* isolated in 2011 from a citrus propagation block grafted with healthy plant material in Réunion Island and (ii) 58 epidemiologically related *X. citri* pv. *citri* isolated in 2006 from three northern provinces in Vietnam (Ha Noi, Hung Yen and Nghe An) [46]. These two additional collections were used to estimate the amount of genetic diversity in well-characterized Asiatic canker outbreaks.

Bacteria were grown on YPGA plates (yeast extract 7 g l^−1^, peptone 7 g l^−1^, glucose 7 g l^−1^ and agar 18 g l^−1^; supplemented with propiconazole 20 mg l^−1^; pH 7.2) at 28°C. Subcultures were used to inoculate YP broth tubes (yeast extract, 7 g l^−1^; peptone 7 g l^−1^; pH 7.2), which were incubated at 28°C on an orbital shaker for 16 to 18 h. Pelleted bacterial cells were used for DNA extraction using the Wizard genomic DNA purification kit (Promega, Charbonnières, France) following the manufacturer's instructions. DNA concentrations were estimated using a Nanodrop ND8000 spectrophotometer (Thermo Fisher, Illkirch, France). Two independent DNA extractions were obtained for each strain and each DNA batch was subsequently genotyped. All strains of *X. citri* pv. *citri* were previously genotyped by AFLP using four selective primer pairs or were AFLP-typed during the present study, as reported earlier [29], [39]. We used AFLP as our genotyping reference method. AFLP was previously found to better describe the genetic variation among pathotypes than insertion sequence or microsatellite-based typing [29].

### VNTR mining from genomic sequences

The IAPAR 306 *X. citri* pv. *citri* genome sequence (Genbank accession number AE008923), seven *X. citri* shotgun genome sequences (pv. *aurantifolii* ACPX01000000 and ACPY01000000, pv. *glycines* AJJO01000000, pv. *malvacearum* AHIB01000000 and AHIC00000000, pv. *mangiferaeindicae* CAHO01000000 and pv. *punicae* CAGJ01000000) were screened using Tandem Repeat Finder in the tandem repeats database for bacteria (http://minisatellites.u-psud.fr) or at http://tandem.bu.edu/trf/trf.html [47], [48]. Parameters were set as follows: the total length in a range of 50–1000 bp, the length of tandem repeats ≥10 bp. Other parameters were set as default. The physical position (kb) of all TR loci in the IAPAR 306 genome was used as a reference (e.g. Xcc0217). Genomic flanking regions (500 bp up- and downstream of the TR loci) were used to define oligonucleotide primer pairs for PCR amplification with the Oligo 6 software (http://www.oligo.net/). Primers were tested by conventional PCR using 20 *X. citri* pv. *citri* strains which served as the “panel test” and represent the currently known genetic diversity within *X. citri* pv. *citri* (Table S1 – data not shown) [27], [29].

### MLVA-31 genotyping

Primer pairs targeting single-locus alleles were used in a multiplex PCR format (multiplex PCR kit, Qiagen, Courtaboeuf, France). One of each primer in the PCR mix was 5′-labeled with one of the following fluorescent dyes: FAM, NED, PET and VIC (Applied Biosystems) (Table 1). PCR reactions contained 5–10 ng of genomic DNA as the template in mixes containing 0.2 to 0.8 µM of each primer, 1× Qiagen multiplex mastermix (containing a hot start *Taq* DNA polymerase), 0.5× Q-solution, and RNase-free water in a total volume of 15 µl. PCR amplifications were performed using the following conditions: 15 min at 95°C for polymerase activation, followed by 25 cycles at 94°C for 30 sec, annealing at temperatures ranging from 64 to 70°C (Table 1) for 90 sec, and 72°C for 90 sec with a final extension step at 72°C for 30 min. One microliter of diluted amplicons (1/10 to 1/50 determined from test runs) was mixed with 0.3 µl of GeneScan-500 LIZ or 0.5 µl of GeneScan-1200 LIZ internal size standard (Applied Biosystems) and 10.7 or 10.5 µl of Hi-Di formamide (for GeneScan-500 LIZ and GeneScan-1200 LIZ, respectively). Capillary electrophoresis was performed in an ABI PRISM-3130xl Genetic Analyzer and results were analyzed with GeneMapper 4.0 (Applied Biosystems). To test the reproducibility of the MLVA-31 technique, two independent DNA extractions were used for all strains, and strain IAPAR 306 of *X. citri* pv. *citri* was used as a control in each experiment.

**Table 1:** Minisatellite and primer description, amplification conditions, number of alleles and Nei's genetic diversity (H_T_) for 31 minisatellite markers tested on strains of *Xanthomonas citri* pv. *citri* from a worldwide strain collection.

Name a	TR length (bp)	ORF b	ORF putative function b	Primers	Annealing temperature (°C)	Primer concentration (µM)	PCR pool	Range of repeat numbers	Number of alleles (H_T_)
Xcc0292	25	−	NA	5′ PET-AGACATCTGCGCAAACGTCC 3′ 5′ CAGCACGGCAGGCGAGCATT 3′	64.0	0.2	6	3–5	3 (0.145)
Xcc0514	205	−	NA	5′ NED-GGCGGAGTTGGCTGGCTAA 3′ 5′ GCGGCGTTGTTTCTGGCATC 3′	68.0	0.2	4	2–3	2 (0.331)
Xcc0677	133	−	NA	5′ NED-ACACCATGGGCGCAGTCAAC 3′ 5′ TGCCGCAGGGAATGGACCGA 3′	70.0	0.6	5	2–4	3 (0.186)
Xcc0724	12	*engXCA*	endoglucanase	5′ PET-CAGCGAGATCGACCAATTGCC 3′ 5′ ATTCTATTGGTCGTGGAACCCC 3′	66.0	0.2	2	4–5	2 (0.471)
Xcc0912	173	−	NA	5′ FAM-ACGACAGAACCCGGCTTATC 3′ 5′ CAGGCGGTGGAAGGGAGT 3′	68.0	0.6	4	1–3	3 (0.183)
Xcc1014	11	+	hypothetical protein	5′ VIC-ATTGCTGCAGTTCCGTCCT 3′ 5′ TCGACCTCTTGCGGTTTCCAG 3′	64.0	0.2	9	4–5	2 (0.031)
Xcc1317	165	−	NA	5′ VIC-TCGGCGATTATGCGTTCTGG 3′ 5′ TTGCGGCTGGCTGTCGTTTG 3′	70.0	0.4	5	2–4	3 (0.131)
Xcc1662	100	+	hypothetical protein	5′ NED-CCTGATTTCGCTTCGTGGTT 3′ 5′ GGCGCTCGTACCATGAG 3′	66.0	0.8	7	6–10	3 (0.031)
Xcc1806	140	+	hypothetical protein	5′ FAM- GAGGCGGCGATGTGGATCA 3′ 5′ CGCGCACCAGACACGGGAGA 3′	70.0	0.2	5	3–6	4 (0.104)
*Xcc1894*	33	+	hypothetical protein	5′ VIC-AGGTTTGAGCAGCGGCCACA 3′ 5′ AAGCACGGGCGCGGTTAT 3′	64.0	0.6	1	2–4	3 (0.507)
*Xcc2059*	117	+	hypothetical protein	5′ PET-TGGAGTTGCGGCAGTCTTGA 3′ 5′ CGGTGGAGCGGTGGGTTA 3′	68.0	0.6	4	1–6	5 (0.499)
*Xcc2072*	18	−	NA	5′ VIC-ACGGCCAACGCATTTCATCTCA 3′ 5′ CCAGCCCACCATCCAGGTCA 3′	66.0	0.2	2	1–4	4 (0.609)
Xcc2229	81	−	NA	5′ NED-CTGCGGTGATCAGGTCCACT 3′ 5′ CCTCCAACGCGATTGC 3′	64.0	0.8	9	5–7	2 (0.031)
Xcc2741	49	−	NA	5′ FAM-CCGGCAAGGAAACTCTGGAT 3′ 5′ GGTGGCGACGCTGGAC 3′	68.0	0.6	3	2–4	3 (0.131)
Xcc2922	186	−	NA	5′ FAM-CGCTGAGTCAGGCAGTCGTT 3′ 5′ GCGTATTGCGGGCGTGTAGG 3′	66.0	0.4	7	3–5	3 (0.075)
Xcc3088	27	−	NA	5′ PET-CTAAGCCTCCGCGCACCAG 3′ 5′ CGCTTGTTGCCGAAAACCGAA 3′	64.0	0.2	6	1–2	2 (0.031)
Xcc3324	26	+	hypothetical protein	5′ VIC-TGATCGAAGCACCGAGCAGT 3′ 5′ GCAACCGGGCAGACCGTTGT 3′	66.0	0.2	8	2–3	2 (0.116)
Xcc3510	24	+	aminopeptidase N	5′ NED-ACCGCTCTACCGAATACGTCA 3′ 5′ ATCGGCATTGTCCATCAACGTC 3′	66.0	0.2	8	1–3	3 (0.031)
*Xcc3522*	10	−	NA	5′ NED-CCCAGCCACCGAACAGATCCG 3′ 5′ AAATCCCTATCGCGCCCAGGT 3′	64.0	0.2	1	2–5	3 (0.075)
*Xcc3816*	18	−	NA	5′ PET-TGGACTGGCTCATGCGTCAG 3′ 5′ ACGAAGGGCTGGGAAT 3′	64.0	0.6	1	2–9	7 (0.706)
*Xcc3993*	12	−	NA	5′ FAM-CGGCGTGGCTGTTCGGTTCC 3′ 5′ AAGACATGGCGAATGCGTCA 3′	64.0	0.2	6	4–8	5 (0.635)
Xcc4071	22	*ggt*	gamma-glutamyltrans-peptidase	5′ FAM-ATTCTCAGTGTCTTAGGGGCCAT 3′ 5′ CGCCGTCCTTCATCACATCCAG 3′	66.0	0.2	8	2–3	2 (0.031)
Xcc4279	33	−	NA	5′ VIC-ATCGGTTCGGCGGCGGTGAT 3′ 5′ AGAAGGGCAGGCGGGCACTC 3′	64.0	0.2	9	2–3	2 (0.015)
*Xcc4322*	27	*styS*	histidine kinase-response regulator hybrid protein	5′ NED-CAAGCACCGGCAGCAAGCGTA 3′ 5′ CGCTGGCCGAGCACTTCCTT 3′	68.0	0.4	3	2–3	2 (0.471)
*Xcc4325*	32	−	NA	5′ FAM-GCCTTGGCGGAACAGACTCA 3′ 5′ TGCCCGTATACGATATGGAT 3′	64.0	0.4	1	1–2	2 (0.439)
*Xcc4372*	158	−	NA	5′ PET-CATGCTGGCGCTGACCTCGTT 3′ 5′ ATTCCCATCTCCCGCCACACC 3′	70.0	0.2	5	1–3	3 (0.522)
*Xcc4424*	15	+	hypothetical protein	5′ PET-CCGAGTTCGCCGACACTGCT 3′ 5′ AGTTTCTTCCACCGCTTCGTCCT 3′	66.0	0.2	2	2–4	3 (0.195)
*Xcc4748*	16	−	NA	5′ PET-GAAGCCCTCAACGCGGTCAA 3′ 5′ CCTCCAACGCGCAATACCGA 3′	68.0	0.4	3	3–13	10 (0.843)
*Xcc4799*	217	*actII-3*	putative xanthomonadin exporter protein	5′ VIC-GACAACGCCATCAGCAGCAG 3′ 5′ CGCCGGTCGTCTCTAAC 3′	68.0	0.6	4	2–4	3 (0.532)
Xcc4927	27	−	NA	5′ FAM-CCCGAGCCAAACCGAATCAC 3′ 5′ GCAGCCGACCCGCGCATCCA 3′	66.0	0.2	8	3–5	3 (0.031)
Xcc4946	30	*aroD*	3-dehydroquinate dehydratase	5′ FAM-CCACAGGCACGCAAGGCCAC 3′ 5′ ACCCATGCCGATCAGGAACTGGA 3′	66.0	0.2	2	2–3	2 (0.015)

a Italicized loci are proposed for routine analyses (MLVA-12).

b As annotated in the IAPAR 306 genome; -: intergenic; NA: not appropriate.

### Data scoring and exploration

Fragment sizes were obtained for each TR locus using GeneMapper 4.0 (Applied Biosystems), transformed to tandem repeat numbers using a conversion table and repeat numbers were used as input data. Repeat numbers of TR arrays with truncated repeats were rounded up to the nearest integer, as recommended [10]. This avoids calling a single truncated copy allele 0, which may be misunderstood as a lack of PCR amplification. The presence and absence of fragments derived from AFLP were scored as a binary matrix from densitograms using GeneMapper 4.0 (Applied Biosystems), as reported previously [29]. Manhattan distances and Dice dissimilarities were computed using the cluster package in R version 2.15.2 (R Core Team, 2012) for MLVA and AFLP data, respectively. A minimum-spanning tree was built using the algorithm recommended for MLVA data combining global optimal eBURST (goeBURST) and Euclidean distances in PHYLOViZ v1.0 [49]. Nei's unbiased estimates of genetic diversity for MLVA-31 data were calculated using ARLEQUIN version 3.01 [50]. Allelic richness (A), the mean number of alleles per locus per population (averaged over all loci), was calculated from MLVA data using the rarefaction procedure for unequal sample sizes (subsample size n = 55) with HP-RARE version 1.0 [51].

The genotypic resolution in relation to the number of TR loci assayed was assessed using two complementary approaches, one of which being similar to that previously implemented on *Mycobacterium tuberculosis* [13]. The first one was based on a sequential concatenation of the most polymorphic loci. Considering n loci, we selected all the combinations of the n most discriminative loci from all the combinations of the (n-1) most discriminative loci and calculated the ratio between the number of haplotypes and the number of strains (G/N). Secondly, a permutation procedure involving a number of draws of n loci among 31 (with n varying from 1 to 31) was implemented to estimate the distribution of the number of discriminated haplotypes for each n. For this, the gtools package version 2.7.1 in R was used. In order to reduce computation time, 1×10^6^ marker combinations were sampled when the total number of combinations exceeded this value. The number of discriminated haplotypes for each class of locus number was estimated by computing minimum, maximum, median and 95% confidence interval values.

The discriminatory power of MLVA-31, MLVA-14 and AFLP was calculated based on Hunter's index (D) [52]. Distance matrices derived from each genotyping technique were compared with a Mantel test using the ‘CADM. post’ functions of ‘ape’ (9999 permutations) in R.

The population structure of the worldwide *X. citri* pv. *citri* strain collection was assessed using Discriminant Analysis of Principal Components (DAPC) [53]. DAPC was used because it is free of any assumption linked to a population genetic model (e.g. Hardy–Weinberg equilibrium, absence of linkage disequilibrium). In DAPC, data transformation is performed by principal component analysis (PCA). Discriminant analysis is conducted using a dataset composed of PCA-transformed, perfectly uncorrelated variables. Using a given number of prior clusters (defined by sequential *k*-means and using the Bayesian information criterion (BIC)), discriminant analysis maximizes the separation between groups while minimizing within-group variation. Twenty independent *k*-means and DAPC runs were performed in order to assess the stability of clustering. One hundred and twenty-nine strains representative of the worldwide genetic and pathological diversity were used (Table S1). Eighty-seven additional bacterial strains originating from North and South America: Argentina (n = 17; isolated from 1977 to 1990), Brazil (n = 62; 1976–2009) and USA Florida (n = 8; 1986–1989) (Table S2), were included in the DAPC analysis as supplementary individuals. This allowed determining to which genetic cluster(s) strains involved in the geographical expansion of *X. citri* pv. *citri* were assigned. Analyses were performed with a single representative strain per haplotype with the ‘adegenet’ package in R. Subclusters were identified within DAPC clusters containing at least 10 haplotypes (i.e. DAPC 1 and DAPC 4). For this, the goeBURST algorithm implemented in PHYLOViZ v1.0 was used [49]. Subclusters were identified as groups of strains linked by up to triple-locus variations.

### MLVA-31 database

A new MLVA website for plant bacterial pathogens, http://www.biopred.net/MLVA/, corresponding to the MLVAbank at http://mlva.u-psud.fr, was created to make MLVA-31 data accessible in an interactive way [54]. The website allows viewing databases with sorting and clustering options, submitting queries, and sharing databases which are maintained and managed by different owners once a common agreement is achieved among partners.

## Results

### Selection of TR markers

Based on the preliminary analysis using the *X. citri* pv. *citri* “panel test” (see Materials & Methods for details), 36 polymorphic TR loci were identified. Five of these were not further considered because (i) Xcc1343, Xcc1742 and Xcc2572 did not provide amplicons for all *X. citri* pv. *citri* strains; (ii) Xcc3912 and its flanking regions were found twice in the IAPAR 306 genome; and (iii) Xcc0699, for which the observed polymorphisms followed 6-bp increments, albeit predicted as an 18 bp-long TR. *In silico* data suggested that the 31 remaining TR loci were largely conserved in other *X. citri* pathovars and displayed size polymorphism among strains. Except for cases corresponding to contig edges, we found that only a single TR locus (Xcc3088) was presumably absent from a single genome (*X. citri* pv. *aurantifolii* accession ACPY01000000). In some cases, Tandem Repeat Finder predicted several alternative TR unit sizes. As a general rule, we chose the TR unit size that best matched our experimental dataset. For example, Xcc1662 was predicted with a TR unit size of 100, 201 or 301 bp. The first option was chosen because we identified a strain from Réunion Island that differed from the sequenced strain IAPAR 306 by a 100-bp increment. Size variations of 100 bp were also identified from *X. citri* shotgun genome sequences. Similarly, Xcc0724 had a predicted TR unit size of 6, 12 or 18 bp. Genotyping data supported by sequence data indicated that only two alleles with a 12-bp size difference are present in the worldwide *X. citri* pv. *citri* collection. The sequenced *X. citri* pv. *citri* strain IAPAR 306 had three complete 12-bp repeat units (plus a truncated one) at Xcc0724, similar to that of other sequenced, genetically related xanthomonads, e.g. *X. citri* pv. *aurantifolii* (ACPX01000194), *X. citri* pv. *glycines* (AJJO01000410), *X. citri* pv. *mangiferaeindicae* (CAHO01000063) or *X. citri* pv. *punicae* (CAGJ01000092). *X. citri* pv. *citri* strains with the alternative allele had four complete 12-bp repeat units (plus a truncated one), similar to *X. axonopodis* pv. *citrumelo* (CP002914), *X. perforans* (AEQW01000199) or some strains of *X. axonopodis* pv. *manihotis* (e.g. AKCZ01000045). Xcc0724 TR is part of the conserved endoglucanase *engXCA* gene of the glycosyl hydrolase 5 (cellulase A) family. It is located in the corresponding C-terminal region of the protein, a region that is not crucial for its activity [55].

### MLVA-31 genotyping

All strains of *X. citri* pv. *citri* could be genotyped at all 31 loci. The assay was highly reproducible since all duplicate tests from independent bacterial cultures of each strain gave fully consistent results. The intra-experimental (within the same run) and inter-experimental (between runs) variation of fragment size calling by capillary electrophoresis typically showed variations of 1 bp when the GeneScan-500 LIZ size standard was used. This permitted unambiguous allele assignation. Standard 2% agarose gel electrophoresis was also performed for all TR loci on selected strains (data not shown). All alleles reported herein could be distinguished when resolved using agarose gels.

**Figure 1:**
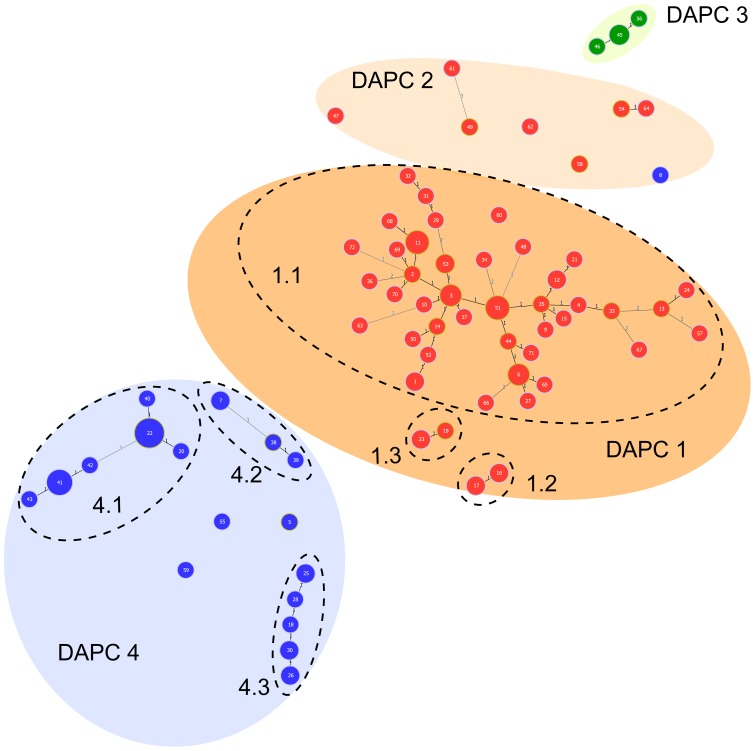
Categorical minimum spanning tree from MLVA-31 data (129 strains –72 haplotypes) representing the genetic diversity within a worldwide strain collection of *Xanthomonas citri* pv. *citri* in relation with its pathological diversity. Dot diameter and color are representative of the number of strains per haplotype and pathotype, respectively (red: pathotype A; blue: pathotype A*; green: pathotype A^w^). Numbers in dots are for haplotype numbers. Numbers along the links indicate the number of polymorphic TR loci distinguishing haplotypes. Haplotypes in a same colored ellipse were assigned to a same genetic cluster by Discriminant Analysis of Principal Components. Dashed ellipses indicate subclusters, as defined by goeBURST [49].

Allele numbers per locus ranged from 2 to 10 (Xcc4748) with 2, 3, 4 and >4 alleles detected for 35, 42, 10 and 13% of the TR loci, respectively. Nei's gene diversity ranged from 0.015 to 0.843 (Table 1). Nine TR loci (Xcc1014, Xcc1662, Xcc2229, Xcc3088, Xcc3510, Xcc4071, Xcc4279, Xcc4927 and Xcc4946) had very low levels of polymorphism and differentiated a single isolate or haplotype (Table 1). Strains sharing an identical MLVA-31 allelic profile always belonged to the same pathotype. A total of 72 MLVA haplotypes among the 129 strains (Table S1) were detected when data from the 31 loci were pooled. The total genetic diversity (H_T_) was 0.263 (0.150 and 0.193 for pathotype A vs. A*/A^w^). MLVA-31 identified 51, 18 and three haplotypes among pathotype A (n = 74), A* (n = 50) and A^w^ (n = 5) strains, respectively. Allelic richness was slightly higher for pathotype A (2.53) than for pathotype A*/A^w^ (2.06). The minimum spanning tree highlighted a clear-cut separation of strains based on their pathotype assignation (A, A* or A^w^) (Fig. 1). We identified very few pathotype-specific single-locus alleles. In contrast with other strains, pathotype A^w^ strains produced a 640-bp amplicon (four TR units) at Xcc1806, as well as a 293-bp or 303-bp amplicon (four or five TR units) at Xcc3522.

### Discriminatory power and evolution of MLVA-31 loci

MLVA-31 was slightly more discriminative than AFLP, with Hunter's *D* values of 0.964 and 0.924, respectively. As expected, both techniques were less discriminative than MLVA-14 (*D* = 0.999). Distance data derived from MLVA-31 and AFLP were strongly positively correlated with a Mantel correlation coefficient of 0.720 (P<0.001). Although significant, Mantel correlation coefficients between MLVA-14 and the two other genotyping techniques were lower (0.500 with AFLP and 0.630 with MLVA-31).

We used the minimum spanning tree of all *X. citri* pv. *citri* strains in order to assess whether a stepwise mutation model (SMM) is a reasonable assumption, as expected for TR markers. Single- (SLV) and double-locus variations (DLV) identified along the evolutionary path of the minimum spanning tree were examined for the associated TR loci and the number of TR repeats involved in the observed polymorphism. Such variations (SLV and DLV) were associated with 15 out of 31 TR loci: Xcc4748 (n = 19 occurrences out of 56, 34%), Xcc3816 (n = 11, 20%), Xcc3993 (n = 5, 9%), Xcc724 (n = 4, 7%), Xcc1894, Xcc4799 (n = 3, 5%), Xcc1662, Xcc2741 (n = 2, 4%), Xcc912, Xcc1317, Xcc2922, Xcc3510, Xcc3522, Xcc4279 and Xcc4927 (n = 1, 2%). TR loci with n>2 occurrences corresponded to loci with a H_T_≥0.45. A total of 86% of these variations (48 out of 56) consisted of single-repeat variations, suggesting that TR loci in the MLVA-31 scheme evolve by stepwise mutations. Multiple-repeat variants, which consisted of double- and triple-repeat variants, primarily corresponded to the most polymorphic loci (e.g. Xcc3816 and Xcc4748). The most likely explanation for their occurrence is the fact that intermediate haplotypes were not represented in our strain collection or that these loci failed to strictly follow a stepwise mutation model. We observed no variations involving gain or loss of >3 repeats, which may be attributed to recombination events [56].

### MLVA-31 cluster analysis

BIC values derived from the *k*-means analysis suggested that four was the lowest appropriate number of prior clusters. The rate of correct re-assignation of individuals to their original clusters (based on discriminant functions), as determined by *k*-means, in the 20 independent runs ranged from 0.986 to 1.000, which suggests very few admixture, if any. All strains but three (JF90-8, LH001-1 and NCPPB 3562) were consistently assigned to a single DAPC cluster in the 20 independent runs with a maximal posterior probability. The three ambiguous strains were most often (i.e. 60% of the runs) assigned to DAPC cluster 2, but also in some runs to DAPC cluster 3 (LH001-1 and NCPPB 3562) or 4 (JF90-8). Increasing *k* yielded a less robust clustering.

The two major DAPC clusters (i.e. DAPC 1 and 4) were further subdivided into subclusters (i.e. networks of strains linked by up to triple-locus variations) using the goeBURST algorithm (Table 2). DAPC cluster 1 was split into three subclusters and one singleton. All bacterial strains assigned to DAPC cluster 1 had a wide host range (i.e. pathotype A). Strains identified as subcluster 1.1 originated from very diverse geographical origins (Southeast Asia, West Asia, Southwest Indian Ocean region, Oceania, North America, South America), whereas strains identified as subclusters 1.2 and 1.3 were geographically restricted to Maldives Islands and Pakistan, respectively. All additional strains from the New World were assigned to DAPC cluster 1 subcluster 1.1 (data not shown). Ninety-four % of these strains were assigned either to haplotype 51 (i.e. the most frequent haplotype) or were SLV of this haplotype. DAPC cluster 2 contained strains originating from West Asia (Bangladesh, India, Oman and Pakistan) and was primarily assigned to pathotype A (Table 2). The single exception was the pathotype A* strain JF90-8 from Oman. This finding was fully consistent with previous AFLP data [27]. Two additional A* strains from India formed a tight cluster with JF90-8 by AFLP and these three strains shared a single MLVA-31 haplotype (data not shown).

**Table 2:** Genetic and pathological diversity and geographical origin of *Xanthomonas citri* pv. *citri* strains within the four DAPC clusters.

	DAPC 1			DAPC 2	DAPC 3	DAPC 4		
N a		67		8	5		49	
N_H_		44		8	3		17	
H_E_		0.115		0.191	0.023		0.152	
Subcluster b	1.1	1.2	1.3	NA	NA	4.1	4.2	4.3
Pathotype	A	A	A	A (A*) c	A^w^	A*	A*	A*
Distribution	Worldwide	Maldives	Pakistan	Bangladesh India Oman Pakistan	India Florida	Iran S Arabia	Oman S Arabia	Cambodia Thailand

a N number of isolates; N_H_ number of haplotypes; H_E_ within-cluster Nei's genetic diversity; NA not appropriate.

b Subclustering was performed based on clonal complexes identified by goeBURST for DAPC clusters containing at least ten haplotypes (see materials and methods) [49].

c A single pathotype A* strain (JF90-8 from Oman) was assigned to DAPC cluster 2.

Among strains with a restricted host range, pathotype A^w^ and pathotype A* strains grouped in DAPC cluster 3 and 4, respectively. DAPC cluster 3 included pathotype A^w^ strains from Florida and India. DAPC cluster 4 was subdivided into three subclusters and three singletons. DAPC subcluster 4.1 contained strains from Iran and Saudi Arabia, DAPC subcluster 4.2 contained strains from Oman and Saudi Arabia, and DAPC subcluster 4.3 contained strains from Cambodia and Thailand (Table 2).

### Analysis of outbreak strains

Outbreak strains from Réunion Island and Vietnam were monomorphic at 30 and 28 (out of 31) TR loci, respectively. The only polymorphic locus for the dataset from Réunion Island was Xcc3816 (H_T_ = 0.706, Table 1) with two alleles consisting of 6 and 7 TR units, yielding two MLVA-31 haplotypes. Using MLVA-14 on the same population (n = 50), polymorphism was observed at eight TR loci and 15 haplotypes were identified. For the dataset from Vietnam (n = 58), we found three polymorphic loci: Xcc4748 (H_T_ = 0.843) with two alleles consisting of 5 and 6 TR units; Xcc3993 (H_T_ = 0.635) with two alleles consisting of 6 and 7 TR units; and Xcc4799 (H_T_ = 0.532) with two alleles consisting of 2 and 3 TR units (Table 1). When genotyped by MLVA-14, these Vietnamese strains were polymorphic at 11 TR loci. The number of MLVA haplotypes was 5 and 55 for MLVA-31 and MLVA-14, respectively. All haplotypes identified among epidemic strains in Réunion Island and Vietnam were assigned to DAPC subcluster 1.1 (data not shown).

### A simplified typing scheme for routine epidemiological investigations

**Figure 2:**
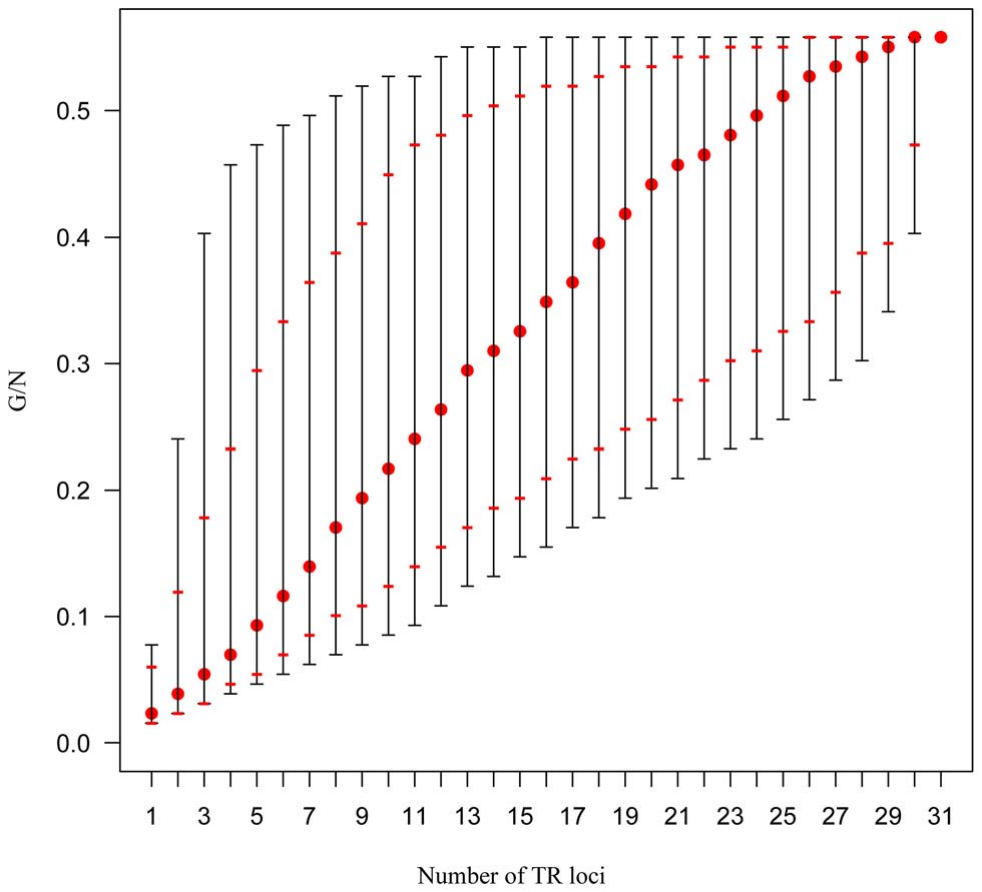
Plot describing the discriminatory power (expressed as G/N, the ratio between the number of haplotypes and the number of strains) in relation to the number of TR loci assayed. Black dashes represent the range of G/N ratios. Red dashes indicate 2.5 and 97.5% quantiles. Red dots indicate the median G/N values.

Based on the sequential approach, a minimum of 14 TR loci was required to discriminate all haplotypes. Permutation tests were performed in order to assess how the ratio between the number of haplotypes and the number of strains (G/N) was influenced by the number of TR loci (Fig. 2). This suggested that a limited number of carefully selected markers could be used for routine analyses. A balanced selection of PCA-uncorrelated, highly polymorphic markers (as suggested by Nei's genetic diversity and their involvement in SLV and DLV in the minimum spanning tree) and markers differentiating genetic lineages (e.g. Xcc3522 and Xcc4424) was made. A genotyping scheme based on 12 TR loci (MLVA-12) (Table 1) that resolved 89% of the total diversity and produced a tree structure consistent with the MLVA-31-based DAPC clustering (Fig. S1) is proposed for routine epidemiological investigations.

## Discussion

Epidemiological surveillance based on genotyping is very useful to assess the geographical expansion of pathogenic bacteria and their prevalence or to monitor newly emerging strains. Due to its numerous technical advantages, MLVA-based genotyping receives more and more attention. The first MLVA scheme for a plant-pathogenic bacterium was developed for *Xylella fastidiosa* in the early 2000s [57]. In 2009, we reported the first MLVA scheme (MLVA-14) for a member of the genus *Xanthomonas*, *X. citri* pv. *citri*, which proved to be useful for analyses at small to medium spatio-temporal scales [44]. Later, MLVA studies on *Clavibacter*, *Erwinia*, *Pseudomonas*, *Ralstonia* and *Xanthomonas* followed [58]–[62].

Until now, no genotyping technique combining discriminatory power and portability was available to assess the global epidemiology of *X. citri* pv. *citri*, a major citrus pathogen worldwide and a quarantine organism in many citrus-producing areas [16]. Here we report a MLVA-based genotyping scheme targeting 31 carefully-selected markers (MLVA-31) for global surveillance of *X. citri* pv. *citri*. MLVA-31 can provide a convenient and powerful way of assigning outbreak strains to *X. citri* pv. *citri* genetic clusters and subclusters by comparing their MLVA profile to data available online. A condensed 12-locus system (MLVA-12) is proposed for routine epidemiological investigations, for instance when a capillary electrophoresis genotyper is not available.

A genotyping technique should appropriately match to the spatio-temporal or evolutionary scale investigated. This has been clearly illustrated for the monomorphic bacterial pathogen *B. anthracis* and led to the development of the ‘progressive hierarchical resolving assays using nucleic acids’ (PHRANA) approach [11]. PHRANA uses a hierarchical progression of diagnostic genomic loci with various levels of evolutionary rate. For *B. anthracis*, the proposed scheme of genotyping techniques consists of (i) single nucleotide polymorphisms (SNP), (ii) MLVA, and (iii) Single Nucleotide Repeats (SNR) [11]. This multi-technique approach has been subsequently applied to other bacterial pathogens (e.g. *Clostridium botulinum* serotype E) [63]. When transposed to *X. citri* pv. *citri*, the MLVA-31 and MLVA-14 methods would correspond to the two latter steps of the hierarchical scheme, respectively [29]. The intended discriminatory power of a genotyping scheme, i.e. according to the investigated scale, can be easily adapted by selecting TRs based on their unit size and total size of the array [8], . Yet, MLVA-based techniques may have limitations for accurately describing deep phylogenetic relationships among lineages. Understanding evolution and population genetics of a monomorphic pathogen, such as *X. citri* pv. *citri*, will require the analysis of nucleotide sequence data obtained from high-throughput genomics [64], [65]. However, also such analyses risk to suffer from phylogenetic discovery bias [4]. Investigating the population biology of *X. citri* pv. *citri* in its native areas is essential, as these areas are likely to host most of the genetic diversity of the pathogen and to constitute a major reservoir for future emerging strains. We are confident that the availability of MLVA-31 and its dedicated online database will allow plant microbiologists to reinforce the epidemiological tracking of *X. citri* pv. *citri* strains and to improve our knowledge on its genetic diversity, especially in native areas.

A comprehensive worldwide collection of *X. citri* pv. *citri* strains was analyzed by Discriminant Analysis of Principal Components (DAPC), as this bacterium shows a strong linkage disequilibrium [29], [46], [53]. Strains were assigned to four DAPC genetic clusters, primarily mirroring the host specialization of the pathogen. West Asia, and more specifically the Indian peninsula (India, Bangladesh and Pakistan), was identified as the region for which the highest genetic diversity was observed as all DAPC clusters and all but one subclusters contained strains originating from this region. Consistently, all singletons in DAPC cluster 1, 2 and 4 also originated from this region. Interestingly, this result was obtained although there is a marked paucity of publicly available microbial resources from this region. Noteworthy, strains with a wide host range among rutaceous species (pathotype A) were separated in two distinct genetic lineages (DAPC 1 and 2). No data comparing the pathogenicity of these two groups of strains are currently available. A single subcluster (1.1) had a very wide geographical range. This suggests that these strains have been primarily implicated in the major geographical expansion of *X. citri* pv. *citri* during the 20^th^ century. We further tested this hypothesis by analyzing 89 additional strains originating from the Americas as supplementary individuals in DAPC and submitting them to goeBURST. All strains were assigned to DAPC 1 subcluster 1.1 (data not shown). In this cluster, strains assigned to the most frequent MLVA-31 haplotypes were isolated from geographically distant areas (including different continents in some cases). This finding suggests recent, long distance migration events of *X. citri* pv. *citri*. The first movements of citrus (in the form of seeds) took place from Asia to the Americas and South Africa in the 1500s, which makes the dispersion of *X. citri* pv. *citri* very unlikely because seed-borne transmission of *X. citri* pv. *citri* is not known to occur so far [19], [66]. By the end of the 19^th^ century, the worldwide expansion of citrus industries and the increasing interest in citrus led to the shipment of large quantities of citrus plants and propagative plant material [67]. Reports describing the biological invasion of *X. citri* pv. *citri* in several American countries suggest that propagative plant material originating from East or South-east Asia was likely to be the primary cause of the long distance spread of *X. citri* pv. *citri* (i.e. DAPC 1 strains) [16], [24], [43]. In contrast, the main natural host species of narrow host range strains (A*/A^w^), Mexican lime, is widely produced from seedlings, at least in many developing countries. The same applies to the rootstock *C. macrophylla* (i.e. the only other host species for which natural infections have been reported for pathotype A*/A^w^ strains). A significant amount of international movement of plant propagative material for these two citrus species occurs through seed. We showed that pathotype A* strains are delineated into several goeBURST subclusters in relation to their geographical origin. We hypothesize that this regional clustering may be explained by the low significance of Mexican lime budwood and whole plants as a pathway for long distance movement of *X. citri* pv. *citri*.

Despite their slight differences in terms of pathogenicity (i.e. Iranian A* strains produce small, non-extensive canker-like lesions on grapefruit and sweet orange, whereas Saudi Arabian A* strains do not), these strains grouped in the 4.1 subcluster, therefore confirming their relatedness, which was shown recently based on their common and unique type III effector repertoire which includes the presence of *xopC1* and *xopAI* in their genomes [27].

The analysis of 58 and 50 epidemiologically related strains of *X. citri* pv. *citri* originating from Vietnam and Réunion Island, respectively, suggested a maximal epidemiological concordance for 28 to 30 out of 31 TR loci [68]. The four variable TR loci (Xcc3816, Xcc3993, Xcc4748 and Xcc4799) were among the most polymorphic loci with relatively high Nei's genetic diversity indices (H_T_>0.5) (Table 1). All the variants for the Vietnam and Réunion Island outbreaks were single-locus and single-repeat variants of the founder haplotype. A single locus (Xcc3816) was polymorphic in the Réunion Island outbreak at a very small spatio-temporal scale (a single citrus nursery block). The Réunion Island outbreak was rapidly identified and controlled through plant destruction; consequently we assume that the strains had little time to evolve. In contrast, three TR loci were polymorphic among strains that were collected in Vietnam during a large ACC outbreak and for which field surveys suggested a possible time frame of approximately five years between the initial outbreak and the sampling, a time frame during which strains possibly underwent microevolution [46]. However, we cannot exclude the possibility that the primary inoculum (probably diseased or contaminated propagative plant material for Vietnam; unknown for Réunion Island) responsible for these ACC outbreaks may have been polyclonal. Such situation is not unlikely to occur given the biological characteristics of the *X. citri* pv. *citri*/citrus pathosystem. A single plant organ (e.g. a twig used as budwood) can host genetically heterogeneous strains. Furthermore, natural spread of *X. citri* pv. *citri* occurs primarily through wind-driven rainwater in which genetically heterogeneous strains may be mixed [16]. Polyclonal primary inoculum has also been reported for other animal and plant pathogens, such as *Listeria monocytogenes*, *Mycoplasma pneumoniae* and *Ralstonia solanacearum* [69]–[71]. A systematic and ongoing typing process will allow estimating the temporal stability of these polymorphic markers. Our study further highlights how MLVA-31 and MLVA-14 schemes produce complementary data to match the spatio-temporal scale and the epidemiological question under investigation.

## Conclusion

The current knowledge of the worldwide population structure of *X. citri* pv. *citri* still remains limited. This is especially the case in its native areas because of the paucity of publicly available microbial resources. Investigating the population biology of monomorphic bacterial plant pathogens in their native areas is important, as native areas constitute a major reservoir for future emerging strains. Here, we presented the characteristics of a new multilocus typing scheme that targets *in silico*-mined minisatellites (MLVA-31) and that allowed to perform a high resolution and robust genotyping of *X. citri* pv. *citri*. This portable and standardized genotyping procedure is combined to an online database that will allow sharing data and comparing new multilocus haplotypes to reference strains. This will contribute to furthering the knowledge of the pathogen's genetic diversity, especially in areas where it is endemic or emerging (e.g. sub-Saharan Africa).

## Supporting Information

Table S1 Worldwide *Xanthomonas citri* pv. *citri* strain collection used in this study.
(DOC)

Table S2 Strains of *Xanthomonas citri* pv. *citri* originating from the New World used as supplementary individuals in the Discriminant Analysis of Principal Components (see Materials & Methods for details).
(DOC)

Figure S1 Categorical minimum spanning tree from MLVA-12 data (129 strains –64 haplotypes) representing the genetic diversity within a worldwide strain collection of *Xanthomonas citri* pv. *citri* in relation with its pathological diversity. Dot diameter and color are representative of the number of strains per haplotype and DAPC cluster, respectively (red: DAPC 1 pathotype A; blue: DAPC 2 pathotype A; light green: DAPC 3 pathotype A^w^; dark green: DAPC 4 pathotype A*). Numbers along the links indicate the number of polymorphic TR loci distinguishing haplotypes.
(TIF)
