# Supplementary material for: A MLVA Genotyping Scheme for Global Surveillance of the Citrus Pathogen Xanthomonas citri pv. citri Suggests a Worldwide Geographical Expansion of a Single Genetic Lineage
Source: PLoS One. 2014 Jun 4;9(6):e98129. doi: 10.1371/journal.pone.0098129 (PMC4045669; doi:10.1371/journal.pone.0098129)
Supplement: Table S1 — Worldwide Xanthomonas citri pv. citri strain collection used in this study. (DOC) [file pone.0098129.s001.doc]

Table S1. Worldwide *Xanthomonas citri* pv*. citri* strain collection used in this study

| Strain number | Pathotype | Country of origin | Host of isolation | Year of isolation | MLVA-31 haplotype number | Genetic cluster (DAPC) |
| --- | --- | --- | --- | --- | --- | --- |
| LG097 | A | Bangladesh | *Citrus limon* | 2006 | 47 | 2 |
| LG098^a^ | A | Bangladesh | *Citrus aurantifolia* | 2006 | 48 | 1 |
| LG099 | A | Bangladesh | *Citrus aurantifolia* | 2006 | 72 | 1 |
| LG102^a^ | A | Bangladesh | *Citrus* sp. | 2006 | 49 | 2 |
| LG111 | A | Bangladesh | *Citrus aurantifolia* | 2006 | 60 | 1 |
| LG113 | A | Bangladesh | *Citrus* sp. | 2006 | 61 | 2 |
| LG114 | A | Bangladesh | *Citrus* sp. | 2006 | 62 | 2 |
| LG117^a^ | A | Bangladesh | *Citrus* sp. | 2009 | 50 | 1 |
| IAPAR306^a^ | A | Brazil | *Citrus sinensis* | 1997 | 6 | 1 |
| JH081-2 | A | China | *Citrus* sp. | 1988 | 9 | 1 |
| JH081-3 | A | China | *Citrus* sp. | 1988 | 10 | 1 |
| JJ035 | A | China | *Citrus tankan* | 1989 | 11 | 1 |
| JJ238-1 | A | China | *Citrus* sp. | NA | 14 | 1 |
| JJ238-2 | A | China | *Citrus* sp. | NA | 51 | 1 |
| JK002-3 | A | China | *Citrus* sp. | NA | 68 | 1 |
| JK002-4 | A | China | *Citrus* sp. | NA | 12 | 1 |
| JK002-8^a^ | A | China | *Citrus* sp. | NA | 21 | 1 |
| JK053 | A | China | *Citrus* sp. | NA | 11 | 1 |
| NCPPB1471 | A | Hong Kong | *Citrus paradisi* | 1963 | 52 | 1 |
| NCPPB211 | A | India | *Citrus* sp. | 1948 | 53 | 1 |
| NCPPB3562^a^ | A | India | *Citrus limon* | 1988 | 54 | 2 |
| NCPPB3610 | A | India | *Poncirus trifoliata* | 1988 | 57 | 1 |
| NCPPB3612 | A | India | *Citrus aurantifolia* | 1988 | 58 | 2 |
| LD107-2 | A | Indonesia | *Citrus* sp. | 2007 | 3 | 1 |
| CFBP2854 | A | Japan | *Citrus* sp. | NA | 51 | 1 |
| CFBP2867 | A | Japan | *Citrus* sp. | 1978 | 4 | 1 |
| CFBP2900 | A | Japan | *Citrus* sp. | NA | 3 | 1 |
| JK146-3 | A | Malaysia | *Citrus reticulata* | 1990 | 34 | 1 |
| JK146-4 | A | Malaysia | *Poncirus trifoliata* | 1990 | 11 | 1 |
| JJ238-10 | A | Maldives islands | *Citrus aurantifolia* | 1987 | 23 | 1 |
| JJ238-11 | A | Maldives islands | *Citrus aurantifolia* | 1987 | 15 | 1 |
| JJ238-8 | A | Maldives islands | *Citrus aurantifolia* | 1987 | 19 | 1 |
| JK004-4 | A | Maldives islands | *Citrus aurantifolia* | 1987 | 23 | 1 |
| CFBP2525 | A | New Zealand | *Citrus limon* | 1957 | 1 | 1 |
| NCPPB226 | A | New Zealand | *Citrus* sp. | 1949 | 1 | 1 |
| JJ238-16 | A | Pakistan | *Citrus sinensis* | 1988 | 16 | 1 |
| JJ238-17 | A | Pakistan | *Citrus sinensis* | 1988 | 17 | 1 |
| JJ238-18 | A | Pakistan | *Citrus sinensis* | 1988 | 16 | 1 |
| JJ238-19 | A | Pakistan | *Citrus sinensis* | 1988 | 17 | 1 |
| JK004-7 | A | Pakistan | *Citrus sinensis* | 1988 | 51 | 1 |
| LH001-1 | A | Pakistan | *Citrus* sp. | 2010 | 64 | 2 |
| JJ223-1 | A | Philippines | *Citrus reticulata* | 1989 | 3 | 1 |
| JK148-2 | A | Philippines | *Citrus sinensis* | 1990 | 37 | 1 |
| JK148-10 | A | Philippines | *Citrus madurensis* | 1990 | 35 | 1 |
| JK148-11 | A | Philippines | *Citrus maxima* | 1990 | 36 | 1 |
| JK148-12 | A | Philippines | *Citrus maxima* | 1990 | 11 | 1 |
| C21 | A | Réunion Island | *Citrus sinensis* | 1987 | 65 | 1 |
| JA143 | A | Réunion Island | *Citrus* sp. | 1981 | 69 | 1 |
| JA167-2 | A | Réunion Island | *Citrus* sp. | 1981 | 67 | 1 |
| JB003-5 | A | Réunion Island | *Citrus aurantifolia* | 1982 | 70 | 1 |
| LB232 | A | South Korea | *Citrus limon* | 2003 | 6 | 1 |
| LB233 | A | South Korea | *Citrus unshiu* | 2003 | 6 | 1 |
| LB234 | A | South Korea | *Citrus natsudaidai* | 2003 | 6 | 1 |
| LB237 | A | South Korea | *Citrus unshiu* | 2004 | 44 | 1 |
| LB238 | A | South Korea | *Citrus limon* | 2004 | 51 | 1 |
| CFBP2548 | A | Taiwan | *Citrus* sp. | NA | 2 | 1 |
| CFBP2549 | A | Taiwan | *Citrus* sp. | NA | 3 | 1 |
| JJ053-7 | A | Taiwan | *Citrus limonia* | 1977 | 12 | 1 |
| JJ053-8 | A | Taiwan | *Citrus jambhiri* | 1977 | 53 | 1 |
| JJ053-9 | A | Taiwan | *Citrus aurantifolia* | 1977 | 11 | 1 |
| JJ166 | A | Taiwan | *Citrus aurantifolia* | NA | 51 | 1 |
| JJ036-1a | A | Thailand | *Citrus reticulata* | 1989 | 11 | 1 |
| JJ036-2 | A | Thailand | *Citrus maxima* | 1989 | 71 | 1 |
| JJ238-23 | A | Thailand | *Citrus reticulata* | 1989 | 51 | 1 |
| JK143-4 | A | Thailand | *Citrus maxima* | NA | 27 | 1 |
| JK143-7 | A | Thailand | *Citrus aurantifolia* | NA | 29 | 1 |
| JK144-2 | A | Thailand | *Citrus hystrix* | 1990 | 31 | 1 |
| JK144-3^a^ | A | Thailand | *Citrus hystrix* | 1990 | 32 | 1 |
| JK144-4 | A | Thailand | *Citrus reticulata* | 1990 | 33 | 1 |
| LMG9322^a^ | A | USA (Florida) | *Citrus aurantifolia* | 1989 | 51 | 1 |
| LC007-1 | A | Viet Nam | *Citrus maxima* | 2006 | 66 | 1 |
| LC053-1 | A | Viet Nam | *Citrus maxima* | 2006 | 63 | 1 |
| JJ185 | A | Yemen | *Citrus aurantifolia* | 1982 | 13 | 1 |
| JK004-9 | A | Yemen | *Citrus* sp. | 1988 | 24 | 1 |
| NCPPB3608^a^ | A^w^ | India | *Citrus aurantifolia* | 1988 | 56 | 3 |
| LB302 | A^w^ | USA (Florida) | *Citrus* sp. | NA | 45 | 3 |
| LB303 | A^w^ | USA (Florida) | *Citrus* sp. | NA | 45 | 3 |
| LB305^a^ | A^w^ | USA (Florida) | *Citrus* sp. | NA | 46 | 3 |
| LB306 | A^w^ | USA (Florida) | *Citrus* sp. | NA | 45 | 3 |
| LD071A | A* | Cambodia | *Citrus* sp. | 2007 | 30 | 4 |
| NCPPB3607^a^ | A* | India | *Citrus aurantifolia* | 1988 | 55 | 4 |
| NCPPB3615^a^ | A* | India | *Citrus aurantifolia* | 1989 | 59 | 4 |
| JM047-2 | A* | Iran | *Citrus aurantifolia* | 1991 | 41 | 4 |
| JS551 | A* | Iran | *Poncirus trifoliata* | 1997 | 42 | 4 |
| JS552 | A* | Iran | *Citrus sinensis* | 1997 | 41 | 4 |
| JS553 | A* | Iran | *Citrus limon* | 1997 | 41 | 4 |
| JS554 | A* | Iran | *Poncirus trifoliata* | 1997 | 41 | 4 |
| JS555 | A* | Iran | *Citrus paradisi* | 1997 | 41 | 4 |
| JS556 | A* | Iran | *Citrus sinensis* | 1997 | 41 | 4 |
| JS558 | A* | Iran | *Citrus latifolia* | 1997 | 43 | 4 |
| JS559 | A* | Iran | *Citrus latifolia* | 1997 | 41 | 4 |
| JS581 | A* | Iran | *Citrus limon* | 1997 | 41 | 4 |
| JS582 | A* | Iran | *Citrus latifolia* | 1997 | 41 | 4 |
| JS584^a^ | A* | Iran | *Citrus* sp. | 1997 | 41 | 4 |
| JF090-2^a^ | A* | Oman | *Citrus aurantifolia* | 1986 | 7 | 4 |
| JF090-3 | A* | Oman | *Citrus aurantifolia* | 1986 | 7 | 4 |
| JF090-8^a^ | A* | Oman | *Citrus aurantifolia* | 1986 | 8 | 2 |
| CFBP2911^a^ | A* | Pakistan | *Citrus* sp. | 1984 | 5 | 4 |
| JK002-9 | A* | Saudi Arabia | *Citrus aurantifolia* | 1988 | 22 | 4 |
| JK002-10^a^ | A* | Saudi Arabia | *Citrus aurantifolia* | 1988 | 22 | 4 |
| JK002-11 | A* | Saudi Arabia | *Citrus aurantifolia* | 1988 | 22 | 4 |
| JK002-12 | A* | Saudi Arabia | *Citrus aurantifolia* | 1988 | 22 | 4 |
| JK002-13 | A* | Saudi Arabia | *Citrus aurantifolia* | 1988 | 22 | 4 |
| JK002-14 | A* | Saudi Arabia | *Citrus aurantifolia* | 1988 | 22 | 4 |
| JK002-16 | A* | Saudi Arabia | *Citrus aurantifolia* | 1988 | 22 | 4 |
| JK002-17 | A* | Saudi Arabia | *Citrus aurantifolia* | 1988 | 22 | 4 |
| JK002-18 | A* | Saudi Arabia | *Citrus aurantifolia* | 1988 | 22 | 4 |
| JK002-19 | A* | Saudi Arabia | *Citrus aurantifolia* | 1988 | 20 | 4 |
| JK002-20 | A* | Saudi Arabia | *Citrus aurantifolia* | 1988 | 22 | 4 |
| JK002-21 | A* | Saudi Arabia | *Citrus aurantifolia* | 1988 | 22 | 4 |
| JK002-22 | A* | Saudi Arabia | *Citrus aurantifolia* | 1988 | 22 | 4 |
| JK002-23 | A* | Saudi Arabia | *Citrus aurantifolia* | 1988 | 22 | 4 |
| JK046 | A* | Saudi Arabia | *Citrus aurantifolia* | 1988 | 22 | 4 |
| JK047 | A* | Saudi Arabia | *Citrus aurantifolia* | 1988 | 22 | 4 |
| JK048 | A* | Saudi Arabia | *Citrus aurantifolia* | 1988 | 22 | 4 |
| JK049 | A* | Saudi Arabia | *Citrus aurantifolia* | 1988 | 22 | 4 |
| JK050 | A* | Saudi Arabia | *Citrus aurantifolia* | 1988 | 22 | 4 |
| JK051 | A* | Saudi Arabia | *Citrus aurantifolia* | 1988 | 22 | 4 |
| JM035-1 | A* | Saudi Arabia | *Citrus aurantifolia* | NA | 38 | 4 |
| JM035-2^a^ | A* | Saudi Arabia | *Citrus aurantifolia* | NA | 39 | 4 |
| JM035-3 | A* | Saudi Arabia | *Citrus aurantifolia* | NA | 40 | 4 |
| JM035-4 | A* | Saudi Arabia | *Citrus aurantifolia* | NA | 22 | 4 |
| JJ238-24^a^ | A* | Thailand | *Citrus aurantifolia* | 1989 | 18 | 4 |
| JK143-1 | A* | Thailand | *Citrus aurantifolia* | NA | 25 | 4 |
| JK143-5^a^ | A* | Thailand | *Citrus aurantifolia* | NA | 28 | 4 |
| JK143-9 | A* | Thailand | *Citrus* sp*.* | NA | 30 | 4 |
| JK143-10 | A* | Thailand | *Citrus aurantifolia* | NA | 25 | 4 |
| JK143-11 | A* | Thailand | *Citrus* sp*.* | NA | 26 | 4 |
| JK143-12 | A* | Thailand | *Citrus aurantifolia* | NA | 26 | 4 |

BCCM/LMG: Belgian Coordinated Collections of Micro-organisms, University of Ghent, Belgium; CFBP: Collection Française de Bactéries Phytopathogènes, Angers, France; IAPAR: Instituto Agronômico do Paraná, Londrina PR, Brazil; NCPPB: National Collection of Plant Pathogenic Bacteria, York, UK.

^a^Test panel strains for preliminary primer screening.

TABLE S2. Strains of *Xanthomonas citri* pv*. citri* originating from the New World used as supplementary individuals in the Discriminant Analysis of Principal Components (see Materials & Methods for details).

| Strain number | Pathotype | Country of origin | Host of isolation | Year of isolation | MLVA-31 haplotype number | Genetic cluster (DAPC) |
| --- | --- | --- | --- | --- | --- | --- |
| JJ155 | A | Argentina | *Citrus aurantifolia* | 1977 | 4 | 1 |
| JJ156 | A | Argentina | *Citrus paradisi* | NA | 51 | 1 |
| JJ157, JJ158 | A | Argentina | *Citrus limon* | 1981 | 51 | 1 |
| JK101-1, JK101-2, JK101-3, JK101-4 | A | Argentina | *Citrus paradisi* | 1990 | 3 | 1 |
| JK102-1, JK102-2, JK102-3, JK102-4, JK103-1, JK103-2 | A | Argentina | *Citrus paradisi* | 1990 | 51 | 1 |
| JK104 | A | Argentina | *Citrus sinensis* | 1990 | 51 | 1 |
| JK105-1, JK105-2 | A | Argentina | *Citrus limon* | 1990 | 51 | 1 |
| CFBP2859 | A | Brazil | *Citrus sinensis* | 1981 | 51 | 1 |
| CFBP2860 | A | Brazil | *Citrus aurantifolia* | 1981 | 51 | 1 |
| CFBP2861 | A | Brazil | *Citrus latifolia* | 1981 | 44 | 1 |
| CFBP2862 | A | Brazil | *Citrus aurantifolia* | 1983 | 51 | 1 |
| CFBP2864 | A | Brazil | *Citrus latifolia* | 1980 | 51 | 1 |
| CFBP2865 | A | Brazil | *Citrus aurantifolia* | 1976 | 51 | 1 |
| IAPAR12411 | A | Brazil | *Citrus* sp. | 1999 | 44 | 1 |
| IAPAR12710 | A | Brazil | *Citrus* sp. | 1999 | 51 | 1 |
| IAPAR12778 | A | Brazil | *Citrus* sp. | 2000 | 79 | 1 |
| IAPAR12844 | A | Brazil | *Citrus* sp. | 2000 | 78 | 1 |
| IAPAR12853 | A | Brazil | *Citrus* sp. | 2009 | 3 | 1 |
| IAPAR12858, IAPAR12861, IAPAR12877 | A | Brazil | *Citrus* sp. | 1996 | 51 | 1 |
| IAPAR12969, IAPAR12984 | A | Brazil | *Citrus* sp. | 2001 | 51 | 1 |
| IAPAR12989 | A | Brazil | *Citrus* sp. | 2001 | 76 | 1 |
| IAPAR13016, IAPAR13017 | A | Brazil | *Citrus* sp. | NA | 51 | 1 |
| IBSBF256 | A | Brazil | *Citrus* sp. | 1980 | 74 | 1 |
| IBSBF1350 | A | Brazil | *Citrus aurantifolia* | 1997 | 2 | 1 |
| IBSBF1518 | A | Brazil | *Citrus* sp. | 2000 | 80 | 1 |
| IBSBF1580 | A | Brazil | *Citrus* sp. | 2001 | 51 | 1 |
| IBSBF1667 | A | Brazil | *Citrus* sp. | 2002 | 77 | 1 |
| LA081-1, LA081-23, LA081-30, LA081-49, LA082-6 | A | Brazil | *Citrus* sp. | NA | 44 | 1 |
| LA081-8, LA081-10, LA081-12, LA081-13, LA081-14, LA081-18, LA081-26, LA081-28, LA081-34, LA081-35, LA081-38, LA081-40, LA081-41, LA081-45, LA081-47, LA081-50, LA081-53, LA081-54, LA081-55, LA094-1, LA094-3, LA094-9, LA094-14, LA094-15, LA094-17 | A | Brazil | *Citrus* sp. | NA | 51 | 1 |
| LA094-8 | A | Brazil | *Citrus* sp. | NA | 75 | 1 |
| LA130, LA146, LA156, LA287 | A | Brazil | *Citrus* sp. | 2001 | 77 | 1 |
| LA140 | A | Brazil | *Citrus sinensis* | 2001 | 73 | 1 |
| LA267 | A | Brazil | *Citrus* sp. | 2001 | 44 | 1 |
| LG130 | A | Brazil | *Citrus* sp. | 2009 | 51 | 1 |
| JJ238-29 | A | USA (Florida) | *Citrus sinensis* | 1986 | 81 | 1 |
| JJ238-30 | A | USA (Florida) | *Citrus aurantifolia* | 1986 | 51 | 1 |
| JJ238-34 | A | USA (Florida) | *Citrus paradisi* | 1988 | 51 | 1 |
| JJ238-35, JJ238-37 | A | USA (Florida) | *Citrus* sp. | 1989 | 51 | 1 |
| JJ238-38, JJ238-39, JJ238-40 | A | USA (Florida) | *Citrus* sp. | 1989 | 44 | 1 |
