# Supplementary material for: A MLVA Genotyping Scheme for Global Surveillance of the Citrus Pathogen Xanthomonas citri pv. citri Suggests a Worldwide Geographical Expansion of a Single Genetic Lineage
Source: PLoS One. 2014 Jun 4;9(6):e98129. doi: 10.1371/journal.pone.0098129 (PMC4045669; doi:10.1371/journal.pone.0098129)
Supplement: Table S2 — Strains of Xanthomonas citri pv. citri originating from the New World used as supplementary individuals in the Discriminant Analysis of Principal Components (see Materials & Methods for details). (DOC) [file pone.0098129.s002.doc]

Table S2. Strains of *Xanthomonas citri* pv*. citri* originating from the New World used as supplementary individuals in the Discriminant Analysis of Principal Components (see Materials & Methods for details).

| Strain number | Pathotype | Country of origin | Host of isolation | Year of isolation | MLVA-31 haplotype number | Genetic cluster (DAPC) |
| --- | --- | --- | --- | --- | --- | --- |
| JJ155 | A | Argentina | *Citrus aurantifolia* | 1977 | 4 | 1 |
| JJ156 | A | Argentina | *Citrus paradisi* | NA | 51 | 1 |
| JJ157, JJ158 | A | Argentina | *Citrus limon* | 1981 | 51 | 1 |
| JK101-1, JK101-2, JK101-3, JK101-4 | A | Argentina | *Citrus paradisi* | 1990 | 3 | 1 |
| JK102-1, JK102-2, JK102-3, JK102-4, JK103-1, JK103-2 | A | Argentina | *Citrus paradisi* | 1990 | 51 | 1 |
| JK104 | A | Argentina | *Citrus sinensis* | 1990 | 51 | 1 |
| JK105-1, JK105-2 | A | Argentina | *Citrus limon* | 1990 | 51 | 1 |
| CFBP2859 | A | Brazil | *Citrus sinensis* | 1981 | 51 | 1 |
| CFBP2860 | A | Brazil | *Citrus aurantifolia* | 1981 | 51 | 1 |
| CFBP2861 | A | Brazil | *Citrus latifolia* | 1981 | 44 | 1 |
| CFBP2862 | A | Brazil | *Citrus aurantifolia* | 1983 | 51 | 1 |
| CFBP2864 | A | Brazil | *Citrus latifolia* | 1980 | 51 | 1 |
| CFBP2865 | A | Brazil | *Citrus aurantifolia* | 1976 | 51 | 1 |
| IAPAR12411 | A | Brazil | *Citrus* sp. | 1999 | 44 | 1 |
| IAPAR12710 | A | Brazil | *Citrus* sp. | 1999 | 51 | 1 |
| IAPAR12778 | A | Brazil | *Citrus* sp. | 2000 | 79 | 1 |
| IAPAR12844 | A | Brazil | *Citrus* sp. | 2000 | 78 | 1 |
| IAPAR12853 | A | Brazil | *Citrus* sp. | 2009 | 3 | 1 |
| IAPAR12858, IAPAR12861, IAPAR12877 | A | Brazil | *Citrus* sp. | 1996 | 51 | 1 |
| IAPAR12969, IAPAR12984 | A | Brazil | *Citrus* sp. | 2001 | 51 | 1 |
| IAPAR12989 | A | Brazil | *Citrus* sp. | 2001 | 76 | 1 |
| IAPAR13016, IAPAR13017 | A | Brazil | *Citrus* sp. | NA | 51 | 1 |
| IBSBF256 | A | Brazil | *Citrus* sp. | 1980 | 74 | 1 |
| IBSBF1350 | A | Brazil | *Citrus aurantifolia* | 1997 | 2 | 1 |
| IBSBF1518 | A | Brazil | *Citrus* sp. | 2000 | 80 | 1 |
| IBSBF1580 | A | Brazil | *Citrus* sp. | 2001 | 51 | 1 |
| IBSBF1667 | A | Brazil | *Citrus* sp. | 2002 | 77 | 1 |
| LA081-1, LA081-23, LA081-30, LA081-49, LA082-6 | A | Brazil | *Citrus* sp. | NA | 44 | 1 |
| LA081-8, LA081-10, LA081-12, LA081-13, LA081-14, LA081-18, LA081-26, LA081-28, LA081-34, LA081-35, LA081-38, LA081-40, LA081-41, LA081-45, LA081-47, LA081-50, LA081-53, LA081-54, LA081-55, LA094-1, LA094-3, LA094-9, LA094-14, LA094-15, LA094-17 | A | Brazil | *Citrus* sp. | NA | 51 | 1 |
| LA094-8 | A | Brazil | *Citrus* sp. | NA | 75 | 1 |
| LA130, LA146, LA156, LA287 | A | Brazil | *Citrus* sp. | 2001 | 77 | 1 |
| LA140 | A | Brazil | *Citrus sinensis* | 2001 | 73 | 1 |
| LA267 | A | Brazil | *Citrus* sp. | 2001 | 44 | 1 |
| LG130 | A | Brazil | *Citrus* sp. | 2009 | 51 | 1 |
| JJ238-29 | A | USA (Florida) | *Citrus sinensis* | 1986 | 81 | 1 |
| JJ238-30 | A | USA (Florida) | *Citrus aurantifolia* | 1986 | 51 | 1 |
| JJ238-34 | A | USA (Florida) | *Citrus paradisi* | 1988 | 51 | 1 |
| JJ238-35, JJ238-37 | A | USA (Florida) | *Citrus* sp. | 1989 | 51 | 1 |
| JJ238-38, JJ238-39, JJ238-40 | A | USA (Florida) | *Citrus* sp. | 1989 | 44 | 1 |
