# Supplementary figures and images for: A MLVA Genotyping Scheme for Global Surveillance of the Citrus Pathogen Xanthomonas citri pv. citri Suggests a Worldwide Geographical Expansion of a Single Genetic Lineage
Source: PLoS One. 2014 Jun 4;9(6):e98129. doi: 10.1371/journal.pone.0098129 (PMC4045669; doi:10.1371/journal.pone.0098129)

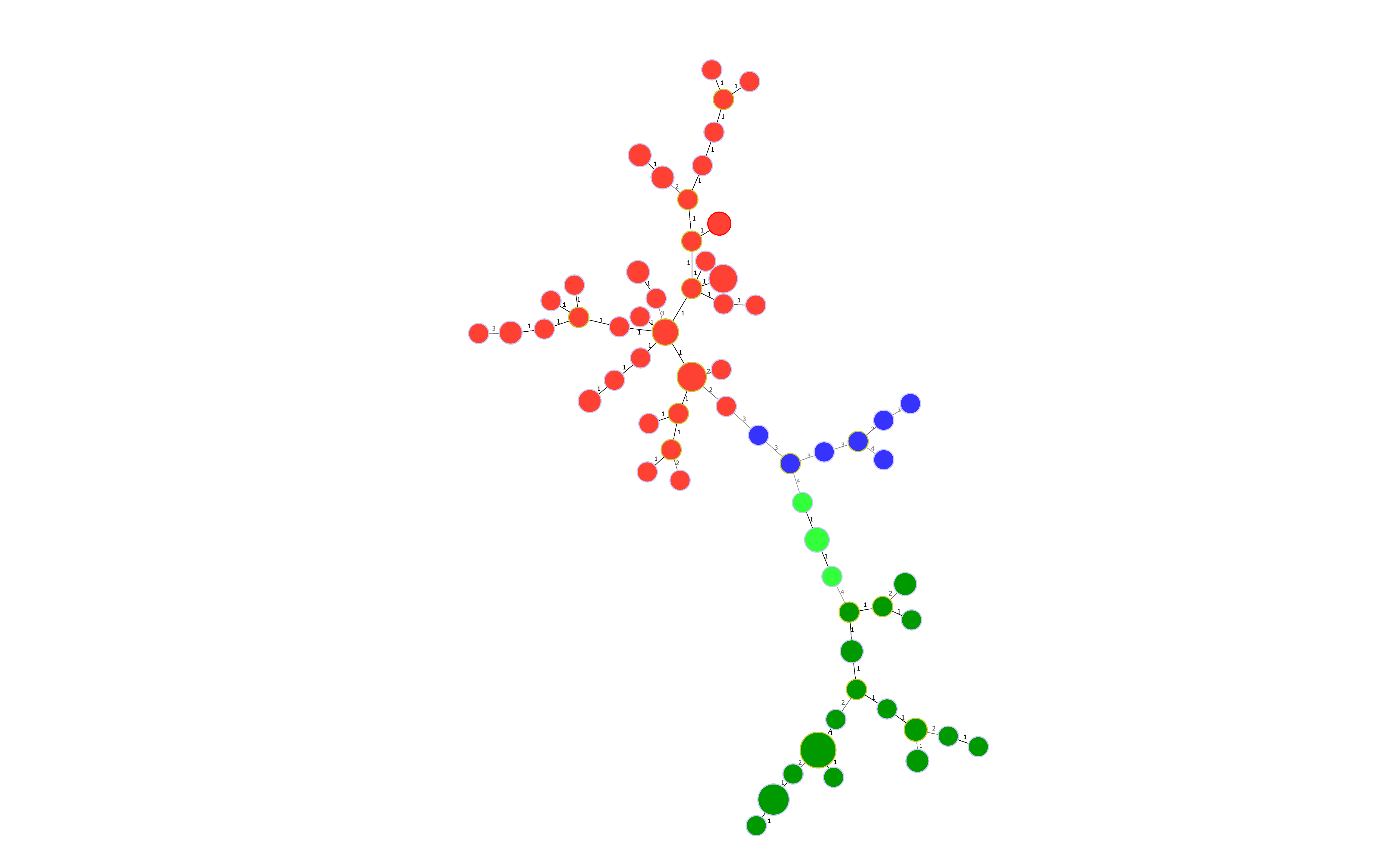

Supplement: Figure S1 — Categorical minimum spanning tree from MLVA-12 data (129 strains –64 haplotypes) representing the genetic diversity within a worldwide strain collection of Xanthomonas citri pv. citri in relation with its pathological diversity. Dot diameter and color are representative of the number of strains per haplotype and DAPC cluster, respectively (red: DAPC 1 pathotype A; blue: DAPC 2 pathotype A; light green: DAPC 3 pathotype Aw; dark green: DAPC 4 pathotype A*). Numbers along the links indicate the number of polymorphic TR loci distinguishing haplotypes. (TIF) [file pone.0098129.s003.tif]
